# Proteorhodopsin Phototrophy Promotes Survival of Marine Bacteria during Starvation

**DOI:** 10.1371/journal.pbio.1000358

**Published:** 2010-04-27

**Authors:** Laura Gómez-Consarnau, Neelam Akram, Kristoffer Lindell, Anders Pedersen, Richard Neutze, Debra L. Milton, José M. González, Jarone Pinhassi

**Affiliations:** 1 Marine Microbiology, School of Natural Sciences, Linnaeus University, Kalmar, Sweden; 2 Department of Molecular Biology, Umeå University, Umeå, Sweden; 3 Department of Chemistry, Biochemistry and Biophysics, Göteborg Gothenburg University, Göteborg, Sweden; 4 Department of Microbiology and Cell Biology, University of La Laguna, La Laguna, Tenerife, Spain; University of California Davis, United States of America

## Abstract

Proteorhodopsins are globally abundant photoproteins found in bacteria in the photic zone of the ocean. Although their function as proton pumps with energy-yielding potential has been demonstrated, the ecological role of proteorhodopsins remains largely unexplored. Here, we report the presence and function of proteorhodopsin in a member of the widespread genus *Vibrio*, uncovered through whole-genome analysis. Phylogenetic analysis suggests that the *Vibrio* strain AND4 obtained proteorhodopsin through lateral gene transfer, which could have modified the ecology of this marine bacterium. We demonstrate an increased long-term survival of AND4 when starved in seawater exposed to light rather than held in darkness. Furthermore, mutational analysis provides the first direct evidence, to our knowledge, linking the proteorhodopsin gene and its biological function in marine bacteria. Thus, proteorhodopsin phototrophy confers a fitness advantage to marine bacteria, representing a novel mechanism for bacterioplankton to endure frequent periods of resource deprivation at the ocean's surface.

## Abstract 2

Mutational analysis provides direct evidence for the link between proteorhodopsin light-harvesting and enhanced survival of marine bacteria.

## Author Summary

It is estimated that marine microscopic algae—phytoplankton—are responsible for half of the Earth's photosynthesis. As much as half of the surface ocean bacteria have proteorhodopsins, which are membrane proteins that allow harvesting of energy from sunlight, implying a potentially significant role of non–chlorophyll-based phototrophy in oceanic carbon cycling and energy flux. Functional evidence for specific roles for proteorhodopsins in native marine bacteria and the marine environment remains surprisingly scarce. One reason for this is the lack of marine bacteria (containing proteorhodopsin genes) that can be maintained in laboratory culture and that are tractable to genetic manipulation. In this study, we show that a proteorhodopsin-containing member of the widespread marine genus *Vibrio* displays light-enhanced survival during starvation in seawater. Furthermore, growth recovery experiments showed that bacteria starving in the light could more rapidly respond to improved growth conditions than those incubated in the dark. We generated a proteorhodopsin deficient *Vibrio* strain and used it to confirm that light-dependent survival of starvation was mediated by the proteorhodopsin. Proteorhodopsin phototrophy thus provides a physiological mechanism that allows surface ocean bacteria to manage an environment where resource availability fluctuates markedly.

## Introduction

Proteorhodopsins (PRs) are membrane-embedded, light-driven proton pumps, which generate a chemiosmotic potential by translocating protons across an energy-transducing membrane [1]–[3]. This proton gradient can subsequently be used for production of biologically available energy in the form of adenosine triphosphate (ATP), the basic energy currency conserved among living beings, and/or for fueling motility and enhancing solute transport across the membrane [2],[3]. The discovery of PR in marine bacteria revealed a possible role of non–chlorophyll-based phototrophy in biogeochemical carbon cycling and energy fluxes in the ocean [1]. Consistent with their inferred ecological importance, PRs are highly abundant and exceedingly genetically diverse in aquatic environments [4]–[10]. The large genetic diversity of PRs suggests that they could potentially display an array of physiological and ecological functions [2],[11],[12]. However, there is a striking lack of knowledge concerning which biological function PRs fulfill and how they contribute to the success of PR-containing bacteria in the marine environment.

Survival and reproduction are the main components that determine fitness, a fundamental concept in ecology. For marine bacteria, the molecular mechanisms that contribute to the variation in these components are poorly understood. Previous studies have focused on the effects of PR on reproduction. In a flavobacterial strain containing PR, light-stimulated growth in seawater was observed [13]. However, similar experiments with other marine bacteria containing PR—Flavobacteria or members of the ubiquitous SAR11 or SAR92 clades—revealed no detectable effect of light on growth [10],[14],[15]. Nevertheless, Lami et al. [16] recently showed that PR expression in SAR11 and Flavobacteria was up-regulated in the presence of light and could be correlated with the abundance of PR genes. Taken together, these findings suggest that PR may involve fitness components other than growth. Thus, in this work, we explored the consequences of PR phototrophy for survival of marine bacteria.

*Vibrio* species are widespread marine bacteria and are frequently referred to as metabolically versatile heterotrophs [17]. Undoubtedly, the most well-known member of the genus is *V. cholerae*, the etiological agent of the disease cholera. Vibrios are typically found associated with detritus particles, algae or zooplankton, as commensals or pathogens on higher organisms, or as free-living populations in the water column [18],[19]. Nevertheless, the potential for phototrophy using PR or other light-harvesting mechanisms has not previously been reported for any member of the genus. We investigated the ecological response to light in a proteorhodopsin-containing member of the genus *Vibrio*. This representative of marine bacteria showed enhanced survival during starvation when exposed to light compared to darkness. Moreover, mutational analysis provided a direct link between the proteorhodopsin gene and the light response that conferred an increased ecological fitness.

## Results/Discussion

**Figure 1:**
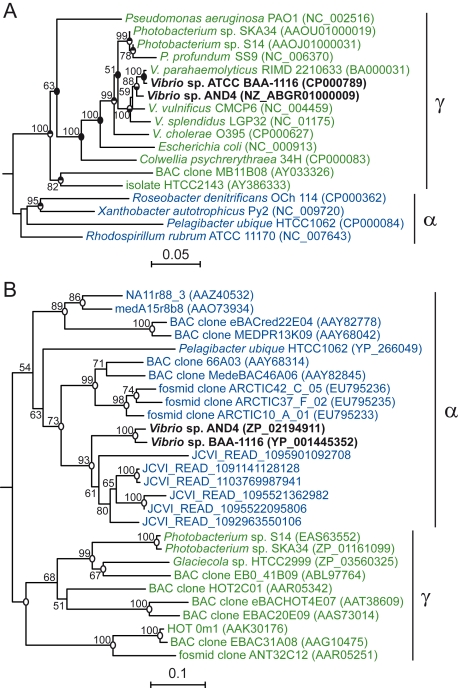
Phylogenetic analyses of 16S rRNA gene and PR peptide sequences. Neighbor-joining trees of (A) 16S rRNA gene, and (B) proteorhodopsin amino acid sequences. *Vibrio* sp. AND4 and *V. harveyi* BAA-1116 sequences (bold face) in relation to sequences of confirmed or tentative Alphaproteobacteria (α; blue) and Gammaproteobacteria (γ; green). The AND4 PR shared sequence similarities between 64% and 68% with PRs of uncultured members of the alphaproteobacterial *Roseobacter* clade, and between 46% and 57% with PRs of Gammaproteobacteria. Numbers at nodes are bootstrap values more than 50% after 1,000 replicates. Circles at nodes, with black filling in the upper or lower half, denote matching topologies in a maximum likelihood tree or a tree generated in ARB, respectively; completely filled circles denote topologies supported by all treeing methods. Scale bars represent substitutions per base position. The divergent ancestry of the 16S rRNA gene and PR peptide sequences suggests that the PR gene of AND4 has been acquired through lateral gene transfer.

A PR-encoding gene was identified from the whole-genome sequence of strain AND4, isolated from surface waters of the Andaman Sea. Phylogenetic analysis of the 16S rRNA gene as well as comparative genome analyses showed that AND4 is a member of the Gammaproteobacteria genus *Vibrio* (Figure 1 and Table 1). The AND4 PR shares a sequence similarity of 87% over 269 amino acid residues with the PR encoded in the publicly available genome sequence of *V. harveyi* strain BAA-1116. To our knowledge, the PR gene has as yet not been found in any other member of the genus *Vibrio*. PRs from the isolates AND4 and BAA-1116 both contain Leu in position 105, which fine tunes the PR light absorption peak towards green light (absorption maximum 535 nm, Figure S1), thereby adapting to the dominant light conditions prevailing in surface seawater [5],[20],[21]. All essential amino acid residues of the energy transducing rhodopsins are conserved (Figure S2), and the protein photocycle has a half-life of approximately 50 ms (Figure S1), as would be expected for a proton pump [22].

**Table 1:** Genome-wide comparison between orthologous peptides in AND4 and other available *Vibrio* genome sequences.

AND4 Compared to	% of Genes Shared^a^ (No. of Orthologs)	Average Peptide Similarity (%)
*V. harveyi* BAA-1116	67.5 (2,889)	90.9
*Vibrio* sp. HY01	40.8 (1,747)	90.5
*V. parahaemolyticus* RIMD2210633	63.0 (2,698)	85.8
*V. parahaemolyticus* AQ3810	54.7 (2,339)	85.5
*V. alginolyticus* 12G01	59.7 (2,554)	85.4
*Vibrio* sp. Ex25	56.2 (2,406)	85.3
*V. vulnificus* YJ016	53.5 (2,291)	77.8
*V. vulnificus* CMCP6	55.4 (2,369)	77.6
*V. splendidus* 12B01	51.0 (2,181)	75.6
*Vibrio* sp. MED222	51.4 (2,198)	75.4
*Vibrio* sp. SWAT-3	52.9 (2,262)	75.0
*V. cholerae* O1 biovar El Tor N16961	49.0 (2,098)	73.4
*V. cholerae* O395	49.7 (2,128)	73.2
*Vibrio shilonii* AK1	50.9 (2,177)	72.1
*V. fischeri* MJ11	50.1 (2,145)	68.3
*V. fischeri* ES114	50.0 (2,139)	68.2

At the time of analysis, 34 genome sequences of *Vibrio* species were publicly available (15 of them being *V. cholerae* strains); included in the table are representatives of the different taxa. Consistent with the phylogenetic analysis of the 16S rRNA gene (Figure 1), genome-wide comparative analyses showed that AND4 was most closely related to *V. harveyi* and *V. parahaemolyticus* also at the genome level, sharing 41%–68% of its putative proteins at a sequence similarity around 86%–91%. The average peptide identity was calculated for pairs of orthologous genes. For each pair of genomes, shared genes were identified based on reciprocal best hits (BLASTP algorithm) with the following settings: E-value<1, percent identity >30%, and coverage >70%. Average amino acid similarities ranging from 93% to 97% mark a boundary for species-level designation [39].

a Calculated as: 100× number of orthologs/number of genes in AND4 (4280 genes).

**Figure 2:**
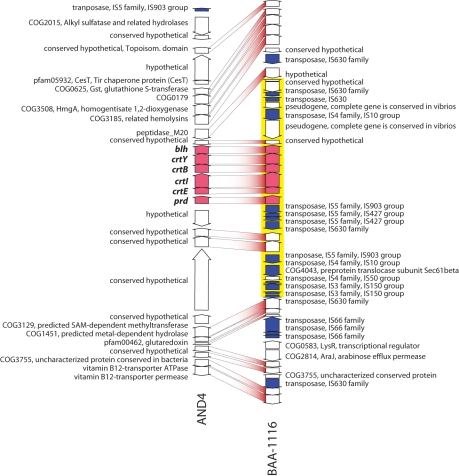
Gene content and organization of PR gene–containing genome segments from AND4 and BAA-1116. PR (*prd*) and chromophore synthesis genes required for functional photoprotein are shown in red, transposase genes in blue. Yellow shading highlights a genomic region predicted by the program IslandPick to have originated from lateral gene transfer [38]. In accordance with the divergent ancestry of the *prd* gene and retinal biosynthesis genes compared to flanking genes (Table S1), the presence of a truncated transposase indicates that the genome region in AND4 containing *prd* has been modified by historical lateral gene transfer events.

In AND4 and BAA-1116, the PR gene and the genes required for synthesis of the chromophore retinal, *crtEIBY* and *blh* [2],[23], were found at the same genetic locus (Figure 2). Phylogenetic analysis of the *Vibrio* PR amino acid sequences showed that, in contrast to the 16S rRNA gene placing AND4 and BAA-1116 among the Gammaproteobacteria, PRs in these bacteria clustered with PRs in Alphaproteobacteria (Figure 1). Moreover, the retinal biosynthesis genes have an ancestry that is divergent from the flanking genes (Table S1). This strongly suggests that the genes for PR and its chromophore have been acquired as a linked set of genes through lateral gene transfer from relatively distantly related bacteria, as has recently been suggested for other marine bacteria [2],. Lateral gene transfer may involve mobile genetic elements since transposase genes are found flanking the PR, *crtEIBY*, and *blh* genes in both BAA-1116 and AND4 (Figure 2). The transposase gene closest to the PR gene in AND4 was truncated and showed best matches to transposases in *V. anguillarum* 775, *V. parahaemolyticus* AQ3776 and *V. cholerae* 91, with percent similarities of 83%–87%. Several of the transposase genes in the genomes of AND4 and BAA-1116 are part of the IS903 subfamily of the IS5 family, which frequently are part of compound transposons in *Vibrio* species [26]. This indicates that AND4 and BAA-1116 share, or have shared, with other vibrios the mechanisms for lateral gene transfer. McCarren and DeLong [25] recently suggested that diverse marine bacteria may have acquired and retained the PR gene because it confers a competitive advantage in an otherwise resource-depleted surface ocean. However, there are no reported studies demonstrating how PR genes that have been putatively acquired through lateral gene transfer could impact on the life strategy of its carrier.

**Figure 3:**
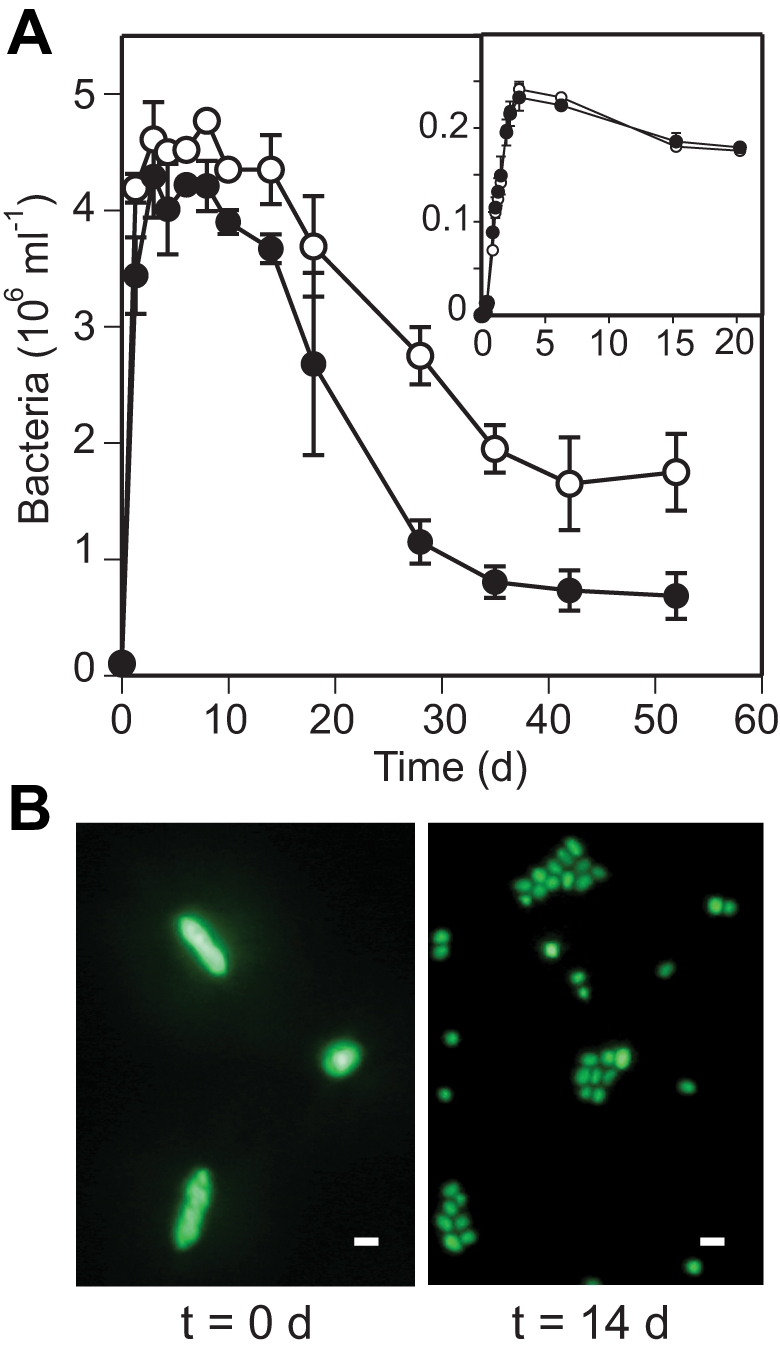
Changes in AND4 cell numbers and morphology in seawater. (A) Bacterial numbers in seawater cultures incubated in the light (open circles) and in the dark (filled circles). Inset: growth in rich medium 8-fold diluted with seawater (for preparation of sterile, particle-free seawater medium, see Materials and Methods); the *x*-axis shows time and the *y*-axis shows optical density at 600 nm. Error bars denote s.d. for duplicate cultures; if not visible, error bars are within symbols. (B) Epifluorescence microscopy images of AND4 cells after 0 d (left) and 14 d (right) incubation in light-exposed seawater. Scale bars indicate 1 µm. The general decrease in bacterial numbers and cell size over time indicates that AND4 bacteria experience starvation in natural seawater. Survival of AND4 in the light was clearly higher compared to dark. AND4 cell numbers in light-exposed seawater remained higher compared to darkness.

To explore this possibility, we investigated the growth and survival of AND4 in light and dark using a suite of approaches, where *light* refers to photosynthetically active radiation. Growth experiments with AND4 in rich medium showed no differences in cell yields for light (continuous light, 133 µmol photons m^−2^ s^−1^) and dark conditions (Figure 3A, inset). Also upon transfer of cells from rich medium to sterile and particle-free natural seawater with low concentrations of organic and inorganic nutrients, an increase of cell numbers within the first 2 d was observed (Figure 3A). Notably, epifluorescence microscopy images of AND4 cultures showed that most of the observed increase in cell numbers was due to reductive division rather than growth, i.e., cell numbers increased, but total biomass did not because each cell decreased in size (Figure 3B). This decrease in cell size is a well-described characteristic of vibrios (and many other bacteria) exposed to starvation, being an important strategy for optimizing cellular energetic efficiency when resources become limited [27]. After 10 d of incubation, bacterial numbers decreased in all cultures, but remained 2.5 times higher in the light compared to darkness. This finding strongly suggests that PR phototrophy can improve the survival of marine bacteria during periods of starvation in seawater.

**Figure 4:**
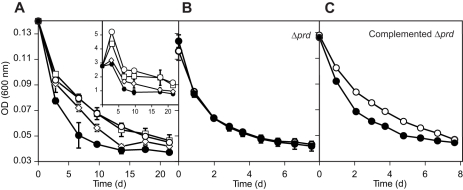
Starvation responses of AND4 exposed to different light conditions. Changes of optical density during starvation of: (A) wild-type AND4 (inset: total bacterial numbers of wild-type AND4 during starvation in seawater; the *y*-axis shows bacteria ml^−1^ [10^8^] and the *x*-axis time in days), (B) Δ*prd* AND4, and (C) Δ*prd* AND4 complemented with the *prd* gene in *trans*. Continuous high-light intensity (open circles), high-light intensity in a 16∶8-h light∶dark cycle (open squares), low-light intensity (open diamonds), or darkness (filled circles). Note different scaling of the *x*-axes. Error bars denote s.d. for duplicate or triplicate cultures; if not visible, error bars are within symbols. These results establish that the PR gene conveys light-enhanced survival during starvation under light conditions equivalent to those found in the surface ocean.

Next, we monitored the development of optical densities (ODs) and bacterial numbers of AND4 grown in rich medium, washed, resuspended in sterile seawater, and exposed to four different light conditions (Figure 4A). In the dark, OD values steeply decreased during the first 13 d of starvation, whereas cultures exposed to light decreased more slowly, with the difference in ODs between the light treatments and darkness changing significantly over time (*F* = 5.81, *df* = 18, 24, *p* = 0.0079). After 7–13 d of starvation, OD values were 40%–60% higher for the treatments with continuous high light and with 16∶8 h light∶dark cycles (133 and 150 µmol photons m^−2^ s^−1^, respectively; corresponding to light intensities in oceanic upper mixed-surface layers), when compared with treatments maintained in the dark. Concomitantly, the effect of light in the low-light treatment (continuous light, at 6 µmol photons m^−2^ s^−1^; corresponding to light intensities at the lower limit of the photic zone) was less pronounced. In parallel with the initial decrease in OD, bacterial numbers peaked on day 3 in the high-light treatments, and thereafter decreased in all treatments. Nevertheless, bacterial numbers remained nearly twice as high for the high-light intensity treatments (Figure 4A, inset). These results again imply that PR can increase the survival rates of bacteria during starvation under light conditions corresponding to those found in the surface ocean.

**Figure 5:**
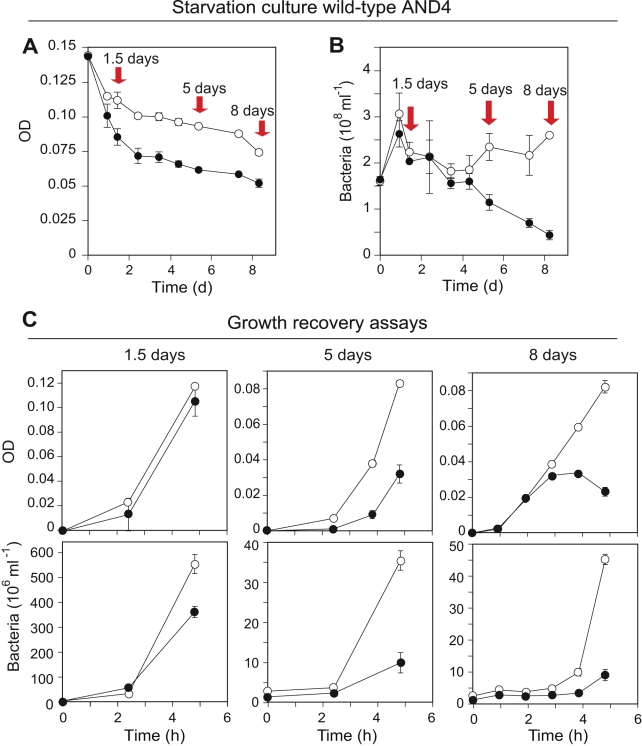
Starvation and growth recovery responses of wild-type AND4. Changes in (A) optical density and (B) total bacterial numbers during starvation in seawater in the light (open circles) or darkness (closed circles). Red arrows indicate the times when recovery experiments in rich medium were performed. (C) Five hours of growth recovery response measured as optical density (upper panels) and total bacterial numbers (lower panels). Note different scaling of the *y*-axes. Error bars denote s.d. for duplicate or triplicate cultures; if not visible, error bars are within symbols. These results show that the PR phototrophy in wild-type AND4 provides: (i) improved survival during starvation when cells are exposed to light, and (ii) improved capacity to recover growth in nutrient-rich environments after starvation in the light.

**Figure 6:**
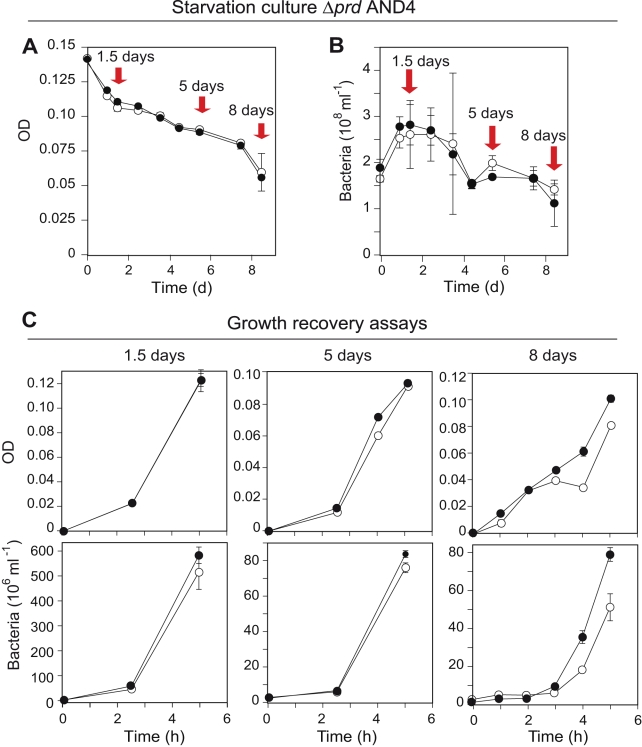
Starvation and growth recovery responses of Δ*prd* AND4. Changes in (A) optical density and (B) total bacterial numbers during starvation in seawater in the light (open circles) or darkness (closed circles). Red arrows indicate the times when recovery experiments in rich medium were performed. (C) Five hours of growth recovery response measured as optical density (upper panels) and total bacterial numbers (lower panels). Note different scaling of the *y*-axes. Error bars denote s.d. for duplicate or triplicate cultures; if not visible, error bars are within symbols. The lack of differences in starvation response and growth recovery between light exposed or dark cultures in Δ*prd* AND4 bacteria confirm that the light-enhanced survival and recovery observed for wild-type AND4 are directly dependent on the presence of the gene encoding PR.

To establish that the PR gene conveys this light-enhanced survival during starvation in AND4, we constructed a strain where the PR gene had been removed by an in-frame deletion of the near-complete PR gene (AND4 Δ*prd*). In contrast with the wild-type behavior (Figures 4A, 5A, and 5B), starvation experiments with the Δ*prd* strain showed no differences in ODs or bacterial numbers between light and dark conditions (Figures 4B, 6A, and 6B). However, when the mutant was complemented with the *prd* gene in *trans*, the wild-type phenotype was regained (Figure 4C; significant day-by-light treatment interaction; *F* = 13.21, *df* = 8, 24, *p* = 0.0083).

Moreover, we analyzed the ability of the wild-type and Δ*prd* strains to recover growth during 5 h incubation in rich medium after increasing periods of starvation (Figures 5 and 6). Although little difference in growth recovery was observed after 1.5 d of starvation, after 5 d, the wild-type bacteria exposed to light during starvation grew to 3- to 6-fold higher densities when compared to bacteria starved in darkness (Figure 5). No differences in recovery were detected in the Δ*prd* strain, irrespective of the history of light-exposed or dark incubations (Figure 6). These growth recovery experiments on wild-type and modified AND4 strains thus confirm that the increased rates of survival and the ability to actively respond to improved growth conditions are a direct consequence of having the light energy–harvesting potential of PR.

Genetic inventories of the world's ocean have revealed that the potential for harvesting light energy by means of PR phototrophy is found in a diverse variety of marine bacteria, encompassing organisms with very different life strategies and physiologies [14],[15],[28]. For example, members of the SAR11 clade are free-living bacteria with a range of cellular and physiological adaptations allowing them to minimize the consequences of starvation in oligotrophic waters. Their PRs are highly expressed, which may be a contributing factor to obtain positive net growth in seawater [14],[16]. For particle-attached bacteria or commensals/pathogens, PR phototrophy could also be important, but on a more irregular basis, during phases of starvation survival between particle or host colonization events. Irrespective of life strategy, the ability to survive starvation while maintaining the potential to proliferate is an essential trait for any evolutionarily successful organism [29].

Our results demonstrate that PR phototrophy represents a physiological mechanism that imparts an improved ability to survive when resources are scarce. This, thus, represents a substantial widening of the phototrophic properties known for marine bacteria in general, and vibrios in particular. *Vibrio* sp. AND4, studied here, is closely related to organisms that are known pathogens on higher organisms (e.g., *V. harveyi* BAA-1116 and *V. parahaemolyticus*) and requires nutrient-rich seawater for growth. AND4 and BAA-1116 are so far the only genome-sequenced members of the genus *Vibrio* that contain the PR gene. For BAA-1116, it is still unknown whether the gene is functional, although the high PR gene region synteny and PR amino acid sequence similarity may suggest that its function is similar to that in AND4. An important challenge for the future will be to unveil the physiological status and growth capacity of other PR-containing bacteria, both cultivated species, and major taxa in the marine environment, and to what extent PR phototrophy may alleviate starvation and/or contribute to other physiological processes in key species. Given the enhanced fitness observed in the present study, the acquisition and maintenance of the PR gene may be highly advantageous in the competitive marine environment, potentially influencing bacterioplankton community composition and population dynamics in the ocean's surface.

## Material and Methods

### Isolation of *Vibrio* sp. AND4

AND4 was isolated from surface water (2-m depth) in the Andaman Sea (7° 48′ 0″ N, 98° 12′ 36″ E) in December 1996 by spreading a 100-µl seawater aliquot on Marine Agar 2216 (Difco). After initial isolation and purification, the isolate was stored in glycerol (20% final concentration) at −70°C.

### Genomic Sequencing and Annotation

Whole-genome sequencing of AND4 was carried out by the J. Craig Venter Institute (JCVI) through the Gordon and Betty Moore Foundation initiative in Marine Microbiology. The draft genome sequence consists of 143 contigs representing ten scaffolds. The genome sequence was obtained using a Sanger/pyrosequencing hybrid method [30]. Our genome analysis is based on open reading frames predicted and annotated using JCVI's prokaryotic annotation pipeline (genome sequence available at https://moore.jcvi.org/moore/). All automatically annotated genes of interest were inspected and verified manually by BLAST, COG, PFAM, and TIGRFAM analyses.

### Data Deposition

The genome sequence (accession no. ABGR00000000; annotation added by the National Center for Biotechnology Information prokaryotic genomes automatic annotation pipeline group), 16S rRNA gene sequence (accession no. AF025960), and proteorhodopsin amino acid sequence (accession no. ZP_02194911) of *Vibrio* sp. AND4 are publicly available in the GenBank database.

### Phylogenetic Trees

For the phylogenetic tree of 16S rRNA genes shown in Figure 1A, a multiple alignment was generated using the software package ClustalW (Version 1.83). The alignment was edited with Gblocks (Version 0.91b) to identify conserved regions. The tree was constructed based on a Jukes-Cantor distance matrix and the Neighbor-Joining method using the PHYLIP package (Version 3.68). The sequence of *Polaribacter* sp. MED152 (DQ481463) served as outgroup. GenBank accession numbers are given in parentheses. The scale bar represents Jukes-Cantor distances (nucleotide substitutions per base position). Filling of circles at nodes represents matching topology with a maximum likelihood tree constructed with RAxML [31] and a neighbor-joining tree (default parameters) constructed with the ARB software package [32].

The proteorhodopsin amino acid sequence tree in Figure 1B was created from a multiple sequence alignment in ClustalW using the PHYLIP software package and the Kimura distance matrix and the neighbor-joining method. The alignment was edited with Gblocks (Version 0.91b) to identify conserved regions with a minimum block of five. The scale bar represents the Kimura distances (number of amino acid substitutions per base position). Circles at nodes represent matching topologies with a maximum likelihood tree constructed with RAxML [31] using the PROTGAMMABLOSUM62F amino acid model. The sequence of *Polaribacter* sp. MED152 (EAQ40925) served as the outgroup.

### Analysis of Transposase Genes

The number of transposase genes was obtained by BLASTP hits against the ISFinder dataset (http://www-is.biotoul.fr) and an E-value<10^−10^ and sequence identity values >35%.

### Functional Measurements on AND4 PR

To obtain measures of AND4 proteorhodopsin absorption maximum and photolysis rates, cell-free expression of AND4 *prd* was carried out by cloning from genomic DNA into the TOPO vector pEXP5NT (Invitrogen) using the sense and antisense primers 5′-ATGAAAAACCAAGTTGAAAAGATAACA-3′ and 5′-TTACGCATCCTGACTCTCGG-3′, respectively. This generated a *prd* construct with an N-terminal 6xhistidine tag followed by a Tev protease cleavage site preceding the AND4 coding sequence. *prd* was expressed with an in-house cell-free expression system based on *Escherichia coli* S12 extract, essentially according to a combination of protocols described in Kim et al. [33] and Torizawa et al. [34]. Expression was performed in batch format at 34°C in the presence of 0.1% Brij35 and 5 µg ml^−1^ all-trans retinal for 2 h at 800 rpm. The resulting reaction mix was centrifuged at 13,000×*g* for 5 min, and the supernatant was bound in batch mode to TALON resin (Clontech) pre-equilibrated with buffer A (phosphate-buffered saline containing 10 mM imidazole and 0.05% Brij35). After 1 h incubation at 4°C, the resin was transferred to a gravity-flow column, washed with 20 column volumes of buffer A, and eluted with buffer B (buffer A with 150 mM imidazole). The resulting eluate was passed through a PD10 column to change the buffer to phosphate-buffered saline containing 0.05% Brij35, and then concentrated with VivaSpin 6 ultrafiltration tubes with a MWCO of 30,000. For flash-photolysis experiments, the buffer was changed to either 25 mM CAPS (pH 10), 0.05% Brij35, or 25 mM MES (pH 5.5), 0.05% Brij35 by repeated dilution and concentration cycles in the ultrafiltration tube.

### Construction of the PR Gene Knockout Mutant of AND4

To create a null mutation in the PR gene, an in-frame deletion was made by allelic exchange using the suicide vector pDM4 as described by Milton et al. [35] with a few minor changes. Plasmid pDM4-rdp-AD, which carries a mutated allele of the PR gene that encodes the first seven amino acids fused to the last eight amino acids of the gene, was introduced into *Vibrio* sp. strain AND4 by conjugation. After the mating, selection for a *Vibrio* strain carrying the plasmid in the chromosome was done using Trypticase soy agar containing 1% sodium chloride, 200 µg ml^−1^ carbenicillin, and 15 µg ml^−1^ chloramphenicol. To complete the allelic exchange, direct selection on Trypticase soy agar containing 5% sucrose for a strain that had lost the *sacB* gene carried on pDM4-rdp-AD was done as described previously. The in-frame deletion was confirmed by sequencing a PCR-amplified DNA fragment of the deleted chromosomal locus. Primers used for the overlap PCR to create the mutated allele were as follows: PR-A-5′-GGACTAGTGGTTACTGGACACAA, PR-B-5′-AACCAAGTTGAAAAGGCAACCTCCGAGAGT, PR-C-5′-CTTTTCAACTTGGTTTTTCATAAT, and PR-D-5′-CTCGAGCTCCAGGGGAGATAGGTT.

To complement the deletion mutation, the wild-type *prd* gene was expressed in *trans* from the plasmid pMMB-prd-wt. To construct pMMB-prd-wt, the *prd* gene was amplified by PCR using KOD polymerase and the primers Rdp-5′-CTCGAGCTCCGTTAAAAGTGAGACTAT and Rdp-3′-CGCGGATCCTGGAAAGAGGGACAGAGA. An 890-bp fragment was gel purified, digested with SacI and BamHI, and ligated to pMMB207 [36], which was similarly digested. The resulting plasmid, pMMB-prd-wt, was mobilized into the Δ*prd* mutant via conjugation.

### Growth, Starvation, and Growth Recovery Experiments

For the rich-medium growth experiment (Figure 3, inset), ZoBell medium (5 g of peptone [Bacto Peptone; BD] and 1 g of yeast extract [Bacto Yeast Extract; Difco] in 800 ml of Skagerrak seawater and 200 ml MilliQ water) was 8-fold diluted in sterile-filtered and autoclaved Skagerrak seawater. Triplicate cultures were incubated at 16°C under an artificial light source of 133 µmol photons m^−2^ s^−1^. Triplicate dark control bottles were covered with aluminum foil. In all experiments, artificial light was provided by fluorescent lamps (L 36W/865, Lumilux, Osram) emitting photosynthetically active radiation, not the full spectrum of sunlight.

For the experiment with natural seawater (Figure 3), water was collected in the Skagerrak Sea and filter sterilized through 0.2 µm-pore-size membrane filters (Supor 200) and autoclaved. Each culture contained 250 ml of seawater (in 500-ml blue-cap glass bottles), and received a final concentration of 100, 2.1, and 0.3 µM dissolved organic carbon (in the form of ZoBell medium), nitrogen (NH_4_Cl), and phosphate (Na_2_HPO_4_), respectively. All material in contact with the samples was acid rinsed with 1 M HCl and extensively washed with MilliQ-water prior to use. Cultures were inoculated with AND4 bacteria previously grown overnight in rich medium (i.e., to early stationary phase). Duplicate light cultures were incubated at 16°C under an artificial light source of 133 µmol photons m^−2^ s^−1^, and duplicate dark controls were covered with aluminum foil.

For the starvation experiments, AND4 cells were grown overnight in 200 ml of rich medium (in 500-ml blue-cap glass bottles). Cells were harvested through centrifugation at 4,000 rpm for 10 min. Cell pellets were washed twice with sterile seawater (filter sterilized through 0.2 µm-pore-size membrane filters and autoclaved), resuspended in seawater, and distributed into Erlenmeyer flasks, with 75 ml of seawater–cells mix in each.

Experiments with the wild-type AND4 included duplicate flasks for the high-light intensity treatment at 133 µmol photons m^−2^ s^−1^, the 16∶8 h light∶dark cycle treatment at 150 µmol photons m^−2^ s^−1^, and the low-light treatment at 6 µmol photons m^−2^ s^−1^. Duplicate dark controls were completely covered with aluminum foil. The experiment was carried out at 16°C. Experiments with the Δ*prd* strain and the Δ*prd* strain complemented with the *prd* gene in *trans* included duplicate or triplicate flasks for each of the strains in the light (continuous light at 133 µmol photons m^−2^ s^−1^) and in dark controls. In a separate experiment, growth recovery experiments with the wild-type and Δ*prd* AND4 strains were performed after 35, 131, and 203 h of starvation in light or darkness. Two hundred microliters of each starved culture were inoculated in 50-ml Falcon tubes that contained 25 ml of ZoBell medium based on Skagerrak seawater. Recovery cultures were incubated at room temperature in the dark and were monitored for 5 h.

Samples for optical density (OD) were measured at 600 nm using a bench top spectrophotometer (Beckman DU 640). Samples for bacterial numbers were fixed with 0.2 µm-pore-size filtered formaldehyde (4%, final concentration), stained with SYBR Gold (1∶100 dilution, Molecular Probes), filtered onto black 0.2 µm-pore-size polycarbonate filters (Poretics, Osmonics Inc.), and counted by epifluorescence microscopy within 48 h. Alternatively, samples for bacterial numbers were fixed with 0.2 µm-pore-size filtered formaldehyde (4%, final concentration), and stored frozen at −70°C until analysis by flow cytometry using a FACSCalibur flow cytometer after staining with Syto13 [37].

### Statistical Analyses

The effect of light on AND4 during starvation was analyzed by repeated-measures analysis of variance. Analyses were performed with PROC GLM in SAS 8.2, using type 3 sums of squares.

## Supporting Information

Figure S1 **Functional measurements on AND4 PR.** pH-dependent absorbance changes of overproduced AND4 PR, with absorbance maxima at 545 (pH 5.5), 535 (pH 7), or 526 (pH 10) nm. Inset: transient absorbance changes at 520 nm (pH 10), following a laser flash at 532 nm. Relaxation to the ground state shows biphasic behavior, with fast and slow half-lives of 35 (57%) and 370 (43%) ms, respectively. The nonlinear least-squares fit to the data is shown as a solid black line. Functional measurements were obtained after cell-free expression of AND4 *prd*.
(0.49 MB EPS)

Figure S2 **Proteorhodopsin amino acid sequences alignment.** Multiple alignment of the predicted amino acid sequences of PR in *Vibrio* sp. AND4 and BAA-1116 compared to other members of Gammaproteobacteria (SAR92 HTCC2207 [ABO88140], *Photobacterium* sp. SKA34 [ZP_01161099], SAR86_blue EBAC20E09 [AAS73014], SAR86_green EBAC31A08 [AAG10475)]), Alphaproteobacteria (SAR11 *Pelagibacter ubique* [AAZ21446]), and *Bacteroidetes* (MED134 [ZP_01049273] and MED152 [ZP_01054176]). Key amino acids for PR functionality are marked by colors: Asp 97 in orange; Gln, Leu, or Met at position 105 in blue or green (in accordance to the amino acid spectral tuning); Glu 108 in red; and Lys 230 in purple (SAR86 eBAC31A08 numbering). Boxes with solid lines mark predicted transmembrane regions. Asterisks mark amino acid positions predicted to be part of the retinal binding pocket. Amino acid sequences were aligned using ClustalW. Conserved amino acids in proton pumping: Lys 216, which binds retinal to helix G through a protonated Schiff base in bacteriorhodopsin, was conserved as Lys 230 in all isolates. Asp 85, the proton acceptor from the Schiff base, was conserved as Asp 97. In marine bacterial PR, Glu 108 replaces Asp 96, which facilitates Schiff-base reprotonation during the latter half of the proteorhodopsin photocycle.
(6.12 MB EPS)

Table S1 **Identity of genome-sequenced organisms containing orthologs to peptides found in the AND4 genome region containing the PR and retinal biosynthesis genes.** The four best matches were retrieved by BLASTP of each AND4 peptide against GenBank. Also shown are the sequence similarity values of the AND4 peptides to the ortholog in each of the best-matching organisms. PR and retinal biosynthesis genes are marked in boldface. Best matches corresponding to non-*Vibrio* Gammaproteobacteria are indicated by the Greek letter “γ” preceding the taxon name, Greek letter “α” denotes Alphaproteobacteria, Greek letter “β” denotes Betaproteobacteria. HTCC2255 is marked by “γ” or “α” depending on whether best-matching peptide is found on assembled contigs belonging to Gamma- or Alphaproteobacteria (the genome sequence derives from at least two different organisms). Note the low sequence similarity values of AND4 peptide orthologs found in non-*Vibrio* genomes compared to those found in other *Vibrio* genomes. *V. parah*. denotes *V. parahaemolyticus*. ND: not detected by the search criteria.
(0.07 MB DOC)

## Abbreviations

OD: optical density
