# Supplementary material for: Proteorhodopsin Phototrophy Promotes Survival of Marine Bacteria during Starvation
Source: PLoS Biol. 2010 Apr 27;8(4):e1000358. doi: 10.1371/journal.pbio.1000358 (PMC2860489; doi:10.1371/journal.pbio.1000358)
Supplement: Table S1 — Identity of genome-sequenced organisms containing orthologs to peptides found in the AND4 genome region containing the PR and retinal biosynthesis genes. The four best matches were retrieved by BLASTP of each AND4 peptide against GenBank. Also shown are the sequence similarity values of the AND4 peptides to the ortholog in each of the best-matching organisms. PR and retinal biosynthesis genes are marked in boldface. Best matches corresponding to non-Vibrio Gammaproteobacteria are indicated by the Greek letter “γ” preceding the taxon name, Greek letter “α” denotes Alphaproteobacteria, Greek letter “β” denotes Betaproteobacteria. HTCC2255 is marked by “γ” or “α” depending on whether best-matching peptide is found on assembled contigs belonging to Gamma- or Alphaproteobacteria (the genome sequence derives from at least two different organisms). Note the low sequence similarity values of AND4 peptide orthologs found in non-Vibrio genomes compared to those found in other Vibrio genomes. V. parah. denotes V. parahaemolyticus. ND: not detected by the search criteria. (0.07 MB DOC) [file pbio.1000358.s003.doc]

**Table S1.**

|  |  | Organisms containing orthologs to AND4 peptides (sequence similarity to ortholog in %) |  |  |  |
| --- | --- | --- | --- | --- | --- |
| AND4 peptide | Gene annotation | Best match | 2^nd^ best match | 3^rd^ best match | 4^th^ best match |
| AND4_02018 | glutathione S-transferase | *V. harveyi* BAA-1116 (84) | *V. harveyi* HY01 (84) | *V. parah.* RIMD 2210633 (73) | *V. splendidus* 12B01 (73) |
| AND4_02023 | conserved hypothetical | *V. harveyi* HY01 (97) | *V. harveyi* BAA-1116 (95) | *Vibrio* sp. Ex25 (92) | *V. alginolyticus* 12G01 (92) |
| AND4_02028 | homogentisate 1,2-dioxygenase | *V. harveyi* HY01 (96) | *V. harveyi* BAA-1116 (96) | *V. parah.* RIMD 2210633 (93) | *Vibrio* sp. Ex25 (92) |
| AND4_02033 | 4-hydroxyphenylpyruvate dioxygenase and related hemolysins | *V. harveyi* HY01 (96) | *V. parah.* RIMD 2210633 (93) | *V. alginolyticus* 12G01 (92) | *V. splendidus* 12B01 (89) |
| AND4_02038 | peptidase M20 | *V. harveyi* BAA-1116 (92) | *V. harveyi* HY01 (92) | *V. parah.* RIMD 2210633 (91) | *V. alginolyticus* 12G01 (87) |
| AND4_02043 | conserved hypothetical | *V. harveyi* BAA-1116 (82) | *V. harveyi* HY01 (80) | *Vibrio* sp. Ex25 (78) | *V. alginolyticus* 12G01 (78) |
| **AND4_02048** | ***blh*** | *V. harveyi* BAA-1116 (62) | ND | ND | ND |
| **AND4_02053** | ***crtY*** | *V. harveyi* BAA-1116 (62) | α; BAL199 (34) | γ; *Marinobacter* sp. ELB17 (30) | β; *R. xylanophilus* DSM 9941 (28) |
| **AND4_02058** | ***crtB*** | *V. harveyi* BAA-1116 (69) | γ; HTCC2207 (44) | α; 66A03 (41) | γ; HTCC2143 (40) |
| **AND4_02063** | ***crtI*** | *V. harveyi* BAA-1116 (82) | γ; HTCC2255 (62) | α; BAL199 (58) | α; HF10_19P19 (57) |
| **AND4_02068** | ***crtE*** | *V. harveyi* BAA-1116 (65) | γ; *Photobacterium* sp. S14 (37) | γ; *Photobacterium* sp. SKA34 (37) | γ; HTCC2255 (34) |
| **AND4_02073** | ***prd*** | *V. harveyi* BAA-1116 (87) | α; 66A03 (70) | α; MedeBAC82F10 (68) | α; HTCC2255 (68) |
| AND4_02078 | hypothetical | ND | ND | ND | ND |
| AND4_02083 | conserved hypothetical | *V. harveyi* BAA-1116 (67) | ND | ND | ND |
| AND4_02088 | conserved hypothetical | *V. harveyi* BAA-1116 (74) | γ; *P. aeruginosa* PA7 (36) | ND | ND |
| AND4_02093 | Mcf protein | *V. harveyi* HY01 (78) | γ; *P. fluorescens* Pf-5 (29) | γ; *P. luminescens* TTO1 (28) | γ; *P. luminescens* TTO1 (28) |
| AND4_02098 | conserved hypothetical | *V. harveyi* HY01 (71) | *Vibrio* sp. SWAT-3 (45) | ND | ND |
| AND4_02103 | conserved hypothetical | *V. harveyi* BAA-1116 (79) | *V. harveyi* HY01 (79) | *Vibrio sp.* DAT722 (78) | *V. alginolyticus* 12G01 (73) |
| AND4_02108 | predicted SAM-dependent methyltransferase | *V. harveyi* HY01 (94) | *V. parah.* AQ3810 (93) | *V. harveyi* BAA-1116 (93) | *Vibrio* sp. DAT722 (93) |
| AND4_02113 | predicted metal-dependent hydrolase | ND | ND | ND | ND |
| AND4_02118 | glutaredoxin | *V. parah.* RIMD 2210633 (94) | *V. alginolyticus* 12G01 (94) | *V. harveyi* BAA-1116 (90) | *V. vulnificus* CMCP6 (85) |
| AND4_02123 | conserved hypothetical | *V. harveyi* HY01 (93) | *V. harveyi* BAA-1116 (92) | *V. parah.* RIMD 2210633 (89) | *V. parah.* AQ3810 (89) |
| AND4_02128 | conserved hypothetical | *V. harveyi* HY01 (98) | *V. harveyi* BAA-1116 (97) | *V. parah.* RIMD 2210633 (95) | *V. alginolyticus* 12G01 (94) |
| AND4_02133 | vitamin B12-transporter ATPase | *V. harveyi* HY01 (89) | *V. harveyi* BAA-1116 (87) | *V. parah.* RIMD 2210633 (82) | *Vibrio* sp. Ex25 (80) |
| AND4_02138 | vitamin B12-transporter permease | *V. harveyi* HY01 (92) | *V. harveyi* BAA-1116 (91) | *V. parah.* AQ3810 (88) | *V. parah.* RIMD 2210633 (88) |
